# Real-world effectiveness and safety of xanomeline and trospium for treatment-resistant schizophrenia in a state hospital system

**DOI:** 10.3389/fpsyt.2025.1736922

**Published:** 2026-01-30

**Authors:** Nina Vadiei, M. Lynn Crismon

**Affiliations:** 1 College of Pharmacy, The University of Texas at Austin, Austin, TX, United States; 2 UT Health San Antonio, San Antonio, TX, United States; 3 San Antonio State Hospital, San Antonio, TX, United States; 4 Department of Psychiatry & Behavioral Sciences, Dell Medical School, The University of Texas at Austin, Austin, TX, United States

**Keywords:** xanomeline and trospium, schizophrenia, treatment-resistant, effective, safety

## Abstract

**Objective:**

Xanomeline and trospium (XT) is a novel medication for schizophrenia that was approved by the United States (U.S.) Food and Drug Administration (FDA) in September 2024. The purpose of this study is to evaluate the effectiveness and safety of using XT in the Texas state hospital setting.

**Methods:**

Data were analyzed retrospectively from five hospitals within the Texas Health and Human Services Commission state hospital system. Adults aged ≥ 18 years administered XT between October 2024 and October 2025 were included. A chart extracted Clinical Global Impression (CGI) of Severity of Illness/Improvement was used to determine XT effectiveness. Patient demographics, clinical characteristics and documented adverse effects are reported.

**Results:**

All patients (N = 20) had treatment-resistant schizophrenia and were classified as markedly or severely ill prior to XT initiation. All patients except one were prescribed ≥ 1 dopamine receptor blocking agents (DRBA)s while taking XT, with olanzapine (n=9; 45%) and clozapine (n=6; 30%) being most common. Fourteen patients (70%) discontinued XT during the study time frame due to intolerability (n=9; 45%) and/or lack of effectiveness (n=12; 60%). The average global improvement CGI score was 4 (no change). Gastrointestinal side-effects were most common, specifically, vomiting (n=9; 45%), dyspepsia (n=5; 25%), and sialorrhea (n=5; 25%).

**Conclusion:**

In inpatients with TRS taking adjunct DRBAs (including anticholinergic DRBAs) XT use was commonly discontinued due to intolerability/ineffectiveness. Larger controlled trials are needed to further investigate XT’s effectiveness for treating TRS and determine how adjunct anticholinergic use impacts its safety/efficacy.

## 1 Introduction

In September 2024, the combination of xanomeline and trospium (XT) was approved by the Food and Drug Administration (FDA) for the treatment of schizophrenia in adults (1). This represents the first FDA approved medication for treating schizophrenia that does not work by functionally blocking dopamine receptors (2). No head-to-head studies comparing XT with dopamine receptor blocking antipsychotics (DRBAs) for the treatment of schizophrenia have been conducted. Additionally, no studies have evaluated the efficacy/safety of using XT in patients with treatment-resistant schizophrenia (TRS). Safe and effective treatments for TRS are urgently needed as 20-50% of patients with schizophrenia do not respond adequately to DRBAs. (3). Furthermore, it has been claimed that as many as 70% of patients with TRS may have dopamine supersensitivty psychosis (4), supporting the need to explore the impact of novel treatments with non-dopaminergic mechanisms of action.

The efficacy and safety of XT for the treatment of schizophrenia in adults was evaluated in short (five-week) randomized, double-blind, placebo-controlled studies (5–10) and an open-label 52-week extension study (11). More data are needed pertaining to its effectiveness and safety in real-world practice (12). Clinicians treating patients with schizophrenia spectrum disorders who are prescribed a DRBA but have only observed minimal/partial response may be interested in starting a medication with a novel mechanism such as XT, but reluctant to discontinue the DRBA due to risk of decompensation. Preclinical research indicates that XT decreases presynaptic dopaminergic activity through multiple mechanisms (13). Furthermore, because XT is an agonist at M_1_ and M_4_ muscarinic receptors, it is unknown how it may interact with those DRBAs that have significant anticholinergic effects. Theoretically, this type of drug-drug interaction may contribute to reduced efficacy and/or tolerability (14). Because of this potential risk, controlled clinical trials investigating the efficacy/safety of XT exclude patients who are concurrently taking other anticholinergic agents. This is evident in studies such as the recent ARISE trial which evaluated XT as an adjunct agent to DRBAs but excluded patients on anticholinergic DRBAs such as olanzapine, quetiapine and clozapine (15).

Despite the unknown effects of combining XT with DRBAs, clinicians may choose to try this combination due to the limited number of pharmacologic agents available for patients with TRS. Thus, it is important to present real-world evidence on how XT is being used in this population that is excluded from clinical trials. This information may help guide clinicians on how to safely and effectively use XT. State hospitals treat a higher proportion of patients with TRS compared to other treatment settings and are therefore an important setting to investigate XT use (16). The purpose of this study was to use real-world data to evaluate the effectiveness/safety of using XT adjunctively with DRBAs in hospitalized patients with TRS.

## 2 Materials and methods

### 2.1 Study design and data source

This study utilized chart data from five Texas state hospitals. Patients in Texas state hospitals are typically admitted for treatment of a serious mental illness (SMI), most commonly schizophrenia spectrum disorders or bipolar disorder. Most patients are ‘forensic’ admissions, meaning they are patients that have either been charged with a crime but necessitate mental health treatment to restore competency to stand trial, or have been deemed ‘Not Guilty by Reason of Insanity (NGRI),’ but require further treatment before being considered safe for community reintegration. These patients tend to have a higher severity of illness and a long history of episodic relapses. Included patients were 18 years or older and prescribed and administered XT between October 2024 and October 2025.

### 2.2 Study variables and definitions

The following data were collected via retrospective chart review: age, sex, race/ethnicity, primary diagnosis, additional charted diagnoses, number of previous DRBA trials in the patient’s lifetime, duration of illness, duration of XT treatment, adjunct medications ordered, vitals, and documented efficacy and adverse effects. Patients were considered treatment-resistant if they had a charted active diagnosis of either schizophrenia or schizoaffective disorder with documentation indicating persistent symptoms despite adequate pharmacologic treatment (defined as > 2 DRBA trials of at least 6 weeks at adequate dose per typical dose ranges outlined in The American Psychiatric Association Practice Guideline for the Treatment of Patients With Schizophrenia) (17). Effectiveness was determined via chart extracted clinical global improvement (CGI) severity of illness and improvement scores, as modeled by a previous study of similar study design (18). Progress notes were reviewed to determine adverse effect reporting while the patient was taking XT. The primary objective was to determine the average CGI improvement score after 6-months of treatment with XT or prior to discontinuing XT (whichever was shorter). The secondary objectives were to determine the incidence of adverse effects when XT was used in combination with other psychotropic agents, including DRBAs and/or additional anticholinergic agents.

### 2.3 Statistical analysis

Descriptive statistics were used for demographic and clinical characteristics. The University of Texas Health San Antonio Institutional Review Board approved the study protocol prior to evaluation of patient data.

## 3 Results

A total of 20 patient records met inclusion criteria and were included for data analysis. Most patients were male (n=18; 90%), White (n=10; 50%) or Black (8; 40%) and were categorized as a forensic admission (n=17; 85%). Most patients were considered markedly or severely ill prior to XT initiation (**Table 1**), and all were considered treatment-resistant. Patients had an average duration of illness of 15 years and an average of five previous DRBA trials prior to XT, including clozapine (n=16; 80%).

**Table 1:** Demographic and clinical characteristics (N = 20).

Variable	Value
Age, mean (SD)	39.1 (10.4)
Duration of illness, years, mean (SD)	15.1 (7.5)
Duration of current admission, years, mean (SD)	2.1 (1.1)
Baseline CGI severity of illness score, mean (SD)	5.5 (0.89)
Number of previous antipsychotic trials, mean (SD)	5.2 (1.7)
Number of scheduled psychotropic medications, mean (SD)	4.6 (2.0)
Total number of scheduled medications, mean (SD)	8.4 (3.8)
Previous clozapine trial, n (%)	16 (80)
Sex, n (%)	
Male	17 (85)
Female	3 (15)
Race/ethnicity, n (%)	
White	10 (50)
Black	8 (40)
Hispanic	2 (10)
Diagnoses, n (%)	
Schizophrenia	4 (20)
Schizoaffective disorder	16 (80)
Treatment-resistant	20 (100)
Personality disorder	1 (5)
Anxiety disorder	0 (0)
Sleep/wake disorder	1 (5)
Neurocognitive disorder	2 (10)
Substance use history	14 (70)
Location, n (%)		
WSH	7 (35)
TSH	7 (35)
RSH	3 (15)
VSH	2 (10)
SAH	1 (5)
Concurrent anticholinergic antipsychotic prescribed (clozapine, olanzapine, quetiapine, chlorpromazine), n (%)	15 (75)
Concurrent psychotropics, n (%)		
Olanzapine	8 (40)
Clozapine	6 (30)
Haloperidol	5 (25)
Paliperidone	5 (25)
Risperidone	4 (20)
Fluphenazine	3 (15)
Aripiprazole	2 (10)
Chlorpromazine	2 (10)
Quetiapine	1 (5)
Lurasidone	1 (5)
Ziprasidone	1 (5)
Valproic acid	9 (45)
Lithium	7 (35)
Lamotrigine	1 (5)
SSRI or SNRI	3 (15)
Other antidepressant	4 (20)
Scheduled benzodiazepine	11 (55)
Other scheduled anticholinergic	7 (35)

SD, Standard deviation; CGI, clinical global impression; WSH, Wichita Falls State Hospital; TSH, Terrell State Hospital; RSH, Rusk State Hospital; VSH, Vernon State Hospital; SAH, San Antonio State Hospital; SSRI, selective serotonin reuptake inhibitor; SNRI, serotonin and norepinephrine reuptake inhibitor.

The median duration of XT treatment was 1.8 months (interquartile range of 1.2 to 2.8 months). All patients but one were prescribed one or more DRBAs while taking XT, with olanzapine (n=8; 40%) and clozapine (n=6; 30%) being most common. Approximately one-third of patients were prescribed an additional scheduled anticholinergic agent (e.g., diphenhydramine or benztropine). Most patients were prescribed scheduled bowel regimens for constipation prophylaxis (n=13; 65%). None of the patients included experienced a clinically significant change in psychotic symptoms during the time they were taking XT (**Table 2**). Fourteen patients (70%) discontinued XT during the study time frame due to intolerability (n=9; 45%) and/or lack of effectiveness (n=12; 60%). While all fourteen patients had no clinically significant change in psychotic symptoms, two of the fourteen were prescribed XT for less than one month. Of the nine patients where tolerability issues were documented as a reason for XT discontinuation, one was prescribed the 50–20 mg dose (5%), six were prescribed the 100–20 mg dose (30%), and two were prescribed the 125–30 mg dose (10%). Two patients were prescribed scheduled medications that inhibit the metabolism of XT (bupropion), though only one of them discontinued XT due to intolerability (on the 100–20 mg dose). Only one patient experienced a possible worsening in symptoms, described as potential behavioral disinhibition.

**Table 2:** Response to xanomeline and trospium (XT) treatment (N = 20).

Efficacy measures	Value
CGI Global Improvement Score, mean (SD)	4.1 (0.2)
Duration of XT treatment, months, median (IQR)	1.8 (1.2-2.8^a^)
Intervention data, n (%)	
Recommended XT titration followed	20 (100)
Max dose reached: 50–20 mg	2 (10)
Max dose reached: 100–20 mg	12 (60)
Max dose reached: 125–30 mg	6 (30)
Tolerability measures, n (%)	
Overall incidence of AEs,	16 (80)
Gastrointestinal AEs	15 (75)
Nausea	6 (30)
Vomiting	9 (45)
Diarrhea	4 (20)
Dyspepsia	5 (25)
Sialorrhea	5 (25)
Constipation	2 (10)
Incontinence	2 (10)
Central nervous system AEs	8 (40)
Dizziness	2 (10)
Sedation	3 (15)
Tremors	1 (5)
Seizure	1 (5)
Dystonia	1 (5)
Autonomic AEs	3 (15)
Tachycardia	3 (15)
Increased BP (> 20/10 mmHg)	2 (10)
Other AEs	3 (15)
Rash	1 (5)
Worsening psychosis	1 (5)
Dental complaints	1 (5)
Discontinuation due to adverse effects	9 (45)
Max dose reached: 50–20 mg	1 (5)
Max dose reached: 100–20 mg	6 (30)
Max dose reached: 125–30 mg	2 (10)
Discontinuation due to inefficacy	14 (70)
Still taking XT at time of chart review	5 (25)

SD, Standard deviation; CGI, clinical global impression; IQR: interquartile range; AE, adverse effect; BP, blood pressure.

a Reported as an interquartile range due to data being nonparametric.

Data pertaining to XT dosing and incidence of adverse effects are detailed in **Table 2**. Gastrointestinal adverse-effects were most common, specifically, vomiting (n=9; 45%), dyspepsia (n=5; 25%), and sialorrhea (n=5; 25%). Constipation was less commonly reported (n=2; 10%). The incidence of cholinergic side-effects was common in patients prescribed concomitant anticholinergic DRBAs (10 of 15; 67%) and non-anticholinergic DRBAs (3 of 4; 75%). The patient that was not prescribed a concomitant DRBA was still taking XT at the time of chart review and had reached the 100–20 mg dose; sedation was the only documented adverse effect. Descriptions of adverse effect reporting and comprehensive adjunct medication orders are available in the **Supplementary Material**.

## 4 Discussion

This is the first study to evaluate the effectiveness and safety/tolerability of XT added to a DRBA in patients with TRS. In this study, patients treated with XT for an average of 2.5 months (median 1.8 months) did not have significant improvement in psychotic symptoms. Furthermore, most patients experienced intolerable adverse effects, with higher incidence rates than previously reported (19, 20).

While many patients experienced adverse effects, with nearly half resulting in XT discontinuation, most documented reasons for XT discontinuation were due to perceived ineffectiveness in treating psychotic symptoms. Recent results from a Phase 3 clinical trial evaluating XT as an adjunctive treatment to nonanticholinergic DRBAs in adults with schizophrenia (ARISE trial) found that adjunctive XT did not reach the threshold for a statistically significant difference compared to adjunctive placebo for the primary endpoint of the change from baseline to Week 6 in the Positive and Negative Syndrome Scale (PANSS) total score. XT in combination with an atypical antipsychotic did however show a numerical improvement compared to treatment with placebo and an atypical antipsychotic in certain patients (15). Specifically, a *post-hoc* subgroup analysis noted a difference in response between subjects treated with risperidone as an adjunct treatment compared with the remaining subjects treated with other atypical antipsychotics (non-risperidone) (15). It is unknown why there was a significant difference between XT used adjunctively with risperidone *vs*. other non-anticholinergic antipsychotics (the non-risperidone group consisted of individuals taking paliperidone, aripiprazole, ziprasidone, lurasidone or cariprazine). Perhaps it may be because of distinct differences in pharmacology; for example, aripiprazole and cariprazine are both partial agonists at the D_2_ receptor and lurasidone and ziprasidone both require adequate calorie consumption to ensure adequate absorption (which can be a difficult administration requirement for patients with schizophrenia treated in the outpatient setting) (17). However, both risperidone and paliperidone have high affinity for dopamine-2 receptors. Regardless, the data were not promising in terms of demonstrating XT effectiveness when used adjunctively with non-anticholinergic DRBAs. This raises the question of how XT used adjunctively with anticholinergic DRBAs may impact treatment response. While data from the current study suggest it is ineffective as an adjunct to DRBAs in patients with TRS, there has been speculation of whether a specific therapeutic approach that takes into account a patient’s unique medication history and current antipsychotic therapy may be necessary to improve the symptoms of schizophrenia with specific types of cholinergic agents (14).

It is particularly important to evaluate the safety of using XT concurrently with more anticholinergic DRBAs, specifically, olanzapine and clozapine. Clozapine is the only antipsychotic FDA-approved for the treatment of TRS and is considered the gold-standard treatment for this population (21). Additionally, some studies suggest that high-dose olanzapine (above the typical high-end of the target dose range) is a viable alternative for patients with TRS that are unable to take clozapine (22, 23). Since these antipsychotics have the highest level of evidence for treatment of TRS, it is not surprising that most patients in the current study sample were taking either olanzapine or clozapine in conjunction with XT, despite these agents being excluded from the original XT clinical trials due to the risk of experiencing additive anticholinergic adverse effects. As previously mentioned, it is possible that combining XT with anticholinergic agents could reduce XT’s efficacy, since its mechanism of action is presumed to be secondary to its agonist activity at M_1_ and M_4_ receptors. One may presume that these potential risks would cause clinicians to avoid using XT in combination with clozapine or olanzapine. This was not the case in this study, as clinicians appeared reluctant to discontinue DRBAs before initiating XT. While it may be a limitation that most patients in the current study were taking concurrent anticholinergic DRBAs, this was the case in this state hospital system. It is unclear whether this is because psychiatric clinicians are unfamiliar with the risks of using XT with anticholinergic DRBAs, or whether it’s perceived that the potential risks are not substantial enough to warrant tapering/discontinuing the DRBA.

It is noteworthy that cholinergic adverse effects such as vomiting, diarrhea, and sialorrhea occurred most often, even in patients that were concurrently using a high burden of anticholinergic medications. However, six of the patients experiencing nausea/vomiting were taking lubiprostone, and nausea has been reported in 17-29% of patients taking lubiprostone for chronic idiopathic constipation (though vomiting is less common) (24). Additionally, most patients that discontinued XT due to reported intolerability had not reached the maximum therapeutic dose. Further research with larger sample sizes in this population are needed to determine whether patients with TRS on multiple psychotropic agents, as well as medications for other disorders, are more likely to develop adverse effects, or if the incidence of certain adverse effects may be higher than reported in clinical trials. It should be noted, however, that the retrospective nature of this study did not allow us to determine with certainty whether documented adverse effects were truly secondary to XT. The **Supplementary Material** details how adverse effects related to XT were reported in the chart and lists all concurrent scheduled medications each patient was taking to provide more context.

XT use requires prior authorization in many health plans due to its current high cost (25). Although criteria vary among health plans, its use may be limited to patients who have failed to respond to one or more DRBAs or are at greater risk of adverse effects from DRBAs. However, it’s important to be mindful that there is currently an absence of evidence supporting the use of XT in patients with TRS. Despite this, clinicians may prescribe new agents with novel mechanisms of action when many alternative options have been tried with minimal to no improvement. Additionally, in patients with TRS who continue to be a danger to themselves/others necessitating ongoing hospitalization, clinicians may be uncomfortable discontinuing DRBAs that have been studied in this population. As such, it’s important to present real-world data on the safety/risks of combining XT with DRBAs, in addition to other medications with potential anticholinergic adverse effects. Until there is more evidence on how concurrent anticholinergic use impacts the efficacy/safety of XT use in TRS, clinicians are advised to take caution using XT in these scenarios.

Most limitations of the current study are inherent to the retrospective study design, lack of a control group, and small sample size. However, given it has been only one year since XT was FDA-approved for the treatment of schizophrenia in adult patients, these data are useful for clinicians considering XT for their patients with TRS. Another potential limitation is that it is difficult to know whether patients in the current sample took XT with or without food, since this is contingent on accurate charting. Not all orders had specific comments communicating the need for nursing staff to ensure XT was administered on an empty stomach. Furthermore, patients on select units are allowed to eat their own snacks outside of scheduled mealtimes, making it difficult for staff to know with absolute certainty whether the patient avoided eating 1 hour prior or 2 hours after XT administration. Of note, findings from a recent open-label study suggest the incidence of adverse effects may be reduced significantly if taken with food (26), despite the current labeling instructing it be taken without food to allow for the sufficient absorption of trospium (20). Similarly, not all adverse effects may have been appropriately documented in the patients’ charts or appropriately assessed/screened for. For example, while constipation was reported less often than other gastrointestinal adverse effects, it is possible that some patients experienced constipation, but it went undetected. Furthermore, most patients were prescribed scheduled bowel regimens for constipation prophylaxis. 

It is also important to note that most patients did not reach the maximum recommended XT dose. A recent case report describes how a patient taking XT initially experienced violent nausea, vomiting and diarrhea on the starting dose of 50 mg/20 mg twice daily, but that this resolved with scheduled ondansetron and reaching the maximum dose of 125 mg/30 mg twice daily (27). The author speculates that the higher dose of trospium may more effectively reduce gastrointestinal side-effects, though this was unclear since the patient was also taking scheduled ondansetron (27). Fixed-dose studies are needed to determine whether certain adverse effects may be dose dependent (28), or whether certain doses have a higher likelihood of achieving treatment response (29). This also raises the question of whether patients on XT experiencing cholinergic adverse effects would benefit from having additional trospium added or having an anti-emetic scheduled for nausea/vomiting prophylaxis. Lastly, findings from this study may not be generalizable to all patients with TRS, considering most patients in the current study sample were forensic admissions in a state hospital setting.

## 5 Conclusion

In this study, patients with TRS taking XT in combination with other DRBAs experienced a high incidence of cholinergic adverse effects and did not experience a clinically significant improvement in symptoms. Larger controlled studies investigating XT for TRS are needed, in addition to comparative studies to further evaluate the clinical impact of taking XT with anticholinergic agents.

## Data Availability

The original contributions presented in the study are included in the article/**Supplementary Material**. Further inquiries can be directed to the corresponding author.
