# Supplementary material for: Real-world effectiveness and safety of xanomeline and trospium for treatment-resistant schizophrenia in a state hospital system
Source: Front Psychiatry. 2026 Jan 30;16:1736922. doi: 10.3389/fpsyt.2025.1736922 (PMC12904147; doi:10.3389/fpsyt.2025.1736922)
Supplement: Supplementary file 1 [file Table1.docx]

**Documented adverse effect reporting (N=20)**

| **Case #** | **Duration of XT treatment (months)** | **Wording used to describe adverse effects with XT** | **Concurrent Prescribed Medications** | **Max dose of XT administered** |
| --- | --- | --- | --- | --- |
| 1 | 4.5 | N/A | Olanzapine 40 mg daily at bedtime  Lithium 300 mg twice daily Sertraline 150 mg at bedtime Clonazepam 1 mg daily at bedtime Fish oil 1g twice daily Docusate-senna 1 tab once daily | 100-20 mg |
| 2 | 1.5 | "Vomited small amount of Gatorade on floor with reported pain to his upper middle back (spine).” *– 9 days after XT initiation, 1 day after titration to 125-30 mg dose.*  "Refused all medications due to reports of N/V/D and stomach pain, dose subsequently lowered.” *– 6 days after titration to 125-30 mg dose, dose subsequently lowered.*  “Patient states ‘the medications are burning my chest and killing it.’” *– 6 days after dose having been lowered, prior to XT discontinuation.* | Fluphenazine decanoate 137.5 mg IM every 2 weeks Olanzapine 30 mg daily at bedtime  Sertraline 100 mg once daily Clonazepam 0.75 mg twice daily and 1 mg every other day Amantadine 100 mg twice daily Metoprolol XL 50 mg once daily | 125-30 mg |
| 3 | 1.5 | “’This medication got me feeling sick. I feel so sick that I don't think I kept it down.’ Says he has been feeling like this the past two days. Denies other physical concerns.” *– 1 week after XT initiation, on 100-20 mg dose.*  “Have attempted to increase this twice but patient had emesis both times. Will hold off on increase for now. Continued to report nausea on low dose, saying ‘I want you to take me off that medication.’”- *5 weeks after XT initiation.* | Haloperidol 20 mg twice daily Olanzapine 40 mg once daily (later tapered and discontinued) Valproic acid 2,000 mg daily  Hydroxyzine pamoate 100 mg once daily at bedtime (and 50 mg daily PRN anxiety) Ondansetron stat order day XT was discontinued | 100-20 mg |
| 4 | 5 | "Patient states he has had diarrhea for about a month…Imodium made it better but not totally well." – *4.5 months after XT initiation, on 125-30 mg dose.* | Aripiprazole 20 mg once daily (discontinued one week after XT initiation) Clozapine 350 mg once daily at bedtime (later switched to olanzapine, then olanzapine switched to risperidone) Bupropion SR 150 mg twice daily Metformin ER 1 g once daily Docusate 200 mg twice daily Lubiprostone 8 mcg twice daily Miralax 17 g once daily | 125-30 mg |
| 5 | 2.25 | "Says he has N/V from the new medication, also says he has heartburn. Says he's only nauseated at night when in bed but feels fine throughout the day. Says he only vomited once.” – *7 days after XT initiation, on 100-20 mg dose.*    “Episode of urinary incontinence,” but this was also noted prior to starting XT. | Clozapine 500 mg once daily ay bedtime Lithium 900 mg once daily at bedtime Venlafaxine XR 75 mg once daily Benztropine 1 mg twice daily (later tapered and discontinued)  Metformin 500 mg twice daily  Calcium carbonate 500 mg once daily Miralax 17 g once daily Lubiprostone 8 mcg twice daily Docusate 100 mg twice daily (later changed to docusate-senna) Docusate-senna 2 tab twice daily Hydroxyzine pamoate 50 mg once daily PRN anxiety | 125-30 mg |
| 6 | 2.25 | “Presents with onset of several episodes of nausea/vomiting this morning; also complains of left pericardial chest pain and some subjective shortness of breath but appears in no respiratory distress."    “Transported to the ER for uncontrolled vomiting and chest pain.” – *1 month after XT initiation, 11 days after titration to 125-30 mg dose.* | Paliperidone 12 mg once daily (later tapered and discontinued)  Quetiapine 400 mg once daily at bedtime  Amantadine 50 mg twice daily Propranolol 10 mg twice daily Terbinafine 250 mg once daily Valsartan 80 mg once daily Docusate 100 mg twice daily Miralax 17 g once daily | 125-30 mg |
| 7 | 3.5 | "Will allow a week before titrating up as last time patient experienced nausea." – *1 week after XT initiation* | Clozapine 400 mg once daily at bedtime (later tapered and discontinued) Lurasidone 60 mg once daily (later tapered and discontinued) Olanzapine 10 mg twice daily (started later and titrated to 30 mg daily) Lithium 900 mg once daily  Docusate-senna 2 tab twice daily Lubiprostone 8 mcg twice daily Bisacodyl 10 mg once daily at bedtime | 125-30 mg |
| 8 | 1 | “Patient had a seizure two days after starting XT (on levetiracetam for seizures).”   “Incontinent of feces, smeared feces on the wall then showered afterwards.” Later that week note reported “patient had dystonic reaction oculogyric crisis requiring IM benztropine.” – *3 weeks after XT initiation, on 100-20 mg dose.*  “Vomited once...had episode of dystonia, observed staring up, woke up with swollen lip and left eye matted shut (said they fell out of bed, laughing while saying this; presumed to have had another seizure).” – *4 weeks after XT initiation, on 100-20 mg dose* | Risperidone 4 mg twice daily Haloperidol 20 mg twice daily Depakote ER 1,250 mg once daily at bedtime Clonazepam 2 mg twice daily Suvorexant 10 mg once daily at bedtime Clonidine 0.1 mg in the morning, 0.2 mg at bedtime Levetiracetam 750 mg twice daily Lubiprostone 24 mcg twice daily Miralax 17 g twice daily Docusate-senna 2 tab twice daily | 100-20 mg |
| 9 | 2^a^ | N/A | Invega Sustenna 234 mg IM once every 4 weeks Risperidone 3 mg twice daily (plan to discontinue after Invega reaches steady state) Quetiapine 100 mg once daily at bedtime (discontinued later) Lamotrigine 75 mg twice daily Lithium 600 mg twice daily Clonazepam 0.5 mg twice daily Eszopiclone 2 mg once daily at bedtime Sertraline 100 mg once daily Trazodone 200 mg once daily at bedtime Amlodipine 10 mg once daily Ferrous sulfate 325 mg twice daily Omeprazole 20 mg once daily Vitamin D3 1,000 mg once daily | 50-20 mg |
| 10 | 9^a^ | “Had a vomiting episode earlier in the morning, patient reports he may have drank his depakene syrup too fast (also on XT 50/20 mg).”   "Reported constipation today. " *– 1 month after XT initiation, on 100-20 mg dose. Abdominal XRAY showed moderate to large volume retained colonic stool and distension of colon.*  "Noted to have increased sialorrhea today." *– 1 month after XT initiation, on 100-20 mg dose.*  "Slight increase in tremor noted today." *–6 months after XT initiation, on 100-20 mg dose.*  “Again transferred to ER for new onset of diaphoresis and lethargy, vomitingx2, dehydration and severe constipation." Lithium level at that time=1.07. Had gone days without a bowel movement necessitating an enema. Abdominal x-ray showed moderate to large volume retained colonic stool and gaseous distension of colon. *– 8 months after XT initiation, on 100-20 mg dose.* | Olanzapine 15 mg twice daily Lithium 300 mg twice daily Clonazepam 0.25 mg twice daily Depakene syrup 500 mg in the morning, 1500 at bedtime Melatonin 9 mg once daily at bedtime  Atropine drops 2 drops SL three times daily Famotidine 20 mg twice daily Folic acid 1 mg once daily Levothyroxine 25 mcg once daily Tamsulosin 0.8 mg once daily at bedtime Lubiprostone 24 mcg twice daily Miralax 17 g twice daily Docusate-senna 2 tab twice daily Quetiapine 200 mg every 8 hours PRN yelling/aggression Dexmedetomidine 120 mcg SL once daily PRN agitation/aggression Dexmedetomidine 60 mcg every two hours PRN agitation/aggression (max 240 mcg per day) | 100-20 mg |
| 11 | 2.25 | “Seen in med clinic over the weekend for constipation and again this morning for emesis.” *– 1.5 months after XT initiation, on 100-20 mg dose.*  "Emesis this morning after breakfast; vomiting and constipation last week." – *1.75 months after XT initiation, on 100-20 mg dose.*  "Complains of stomach upset with medication (XT)." - *2.25 months after XT initiation, on 100-20 mg dose.* | Invega Sustenna 234 mg IM every 4 weeks Loratadine 10 mg once daily Docusate-senna 2 tab twice daily Miralax 17g twice daily (started later) Lubiprostone 24 mcg twice daily (started after med clinic visit)  Bisacodyl 20 mg once daily (started after med clinic visit) | 100-20 mg |
| 12 | 1.25^a^ | "He said it's good but makes him sleepy." – *1 week after XT initiation, on 100-20 mg dose.* | Clonazepam 2 mg twice daily Melatonin 10 mg once daily at bedtime Metoprolol XL 25 mg once daily Vitamin D3 2,000 IU once daily Lubiprostone 24 mcg twice daily Miralax 17g twice daily Docusate-senna 2 tab twice daily Zolpidem 10 mg once daily at bedtime PRN insomnia Haloperidol 5 mg + lorazepam 2 mg PRN agitation every 6 hours PRN agitation | 100-20 mg |
| 13 | 1.5 | “Seborrheic dermatitis of the face. Seen in clinic for complaints of itchy forehead and red patches on his forehead. Patient reports noticing flaking and rash, unable to say how long he had it. Could not get much history from patient.” – *6 days after XT initiation, on 100-20 mg dose.*  "We subsequently tapered it [clozapine] 100 mg/day so as to allow safer titration on XT and to mitigate any anticholinergic burden. However, patient had an exacerbation of psychosis and worsening of sialorrhea, so we discontinued XT and resumed titrating clozapine up." – *1.5 months after XT initiation, on 100-20 mg dose.* | Haloperidol 20 mg twice daily Clozapine 100 mg once daily at bedtime (plan to taper and discontinue) Glycopyrrolate 2 mg twice daily Depakote DR 500 mg twice daily Atropine drops 2 drops SL twice daily | 100-20 mg |
| 14 | 7.5^a^ | "The patient reported N/V due to XT and asked if he should take another medication or switch. After discussing, we agreed to prescribe ondansetron 4 mg BID. The patient appeared calm and composed, greeting everyone with a fist pump before leaving the room today." *– 1 month after XT initiation, on 100-20 mg dose.*  “Dizziness and head spinning…he reported that XT ‘is causing holes in my teeth’ and no longer wishes to take it. *– 4.5 months after XT initiation, on 100-20 mg dose.* Note indicated plan to discontinue XT that day, but it was not discontinued.  “Referred to med clinic. Patient vomited moderate amount of white food particles and was shaking. He thinks it is his XT medication. Patient reports that he has been on XT for a few months now and when XT was started, he started having nausea and hence ondansetron was started along with it. Now he gets XT and ondansetron together before meals, but he says that he has been unable to tolerate XT and is planning to talk to his doctor about discontinuation of the same or switching to a different medication. He reports his dose was recently increased and as a result he had one episode of vomiting about 4 days ago…He reports that although he does occasionally have nausea, he rarely vomits. Consider either lowering the dose of XT or changing it to a different medication." *– 6 months after XT initiation, on 100-20 mg dose.* | Haloperidol 20 mg twice daily Olanzapine 20 mg twice daily (later tapered to 10 mg twice daily) Trazodone 50 mg once daily at bedtime Benztropine 1 mg twice daily Ondansetron ODT 4 mg BID before meals Miralax 17 g once daily | 100-20 mg |
| 15 | 0.1 | "We tried XT but patient reported excessive sialorrhea and requested discontinuation." – *1 day after XT initiation.* | Fluphenazine 20 mg twice daily Chlorpromazine 400 mg twice daily Depakote ER 500 mg once daily at bedtime Clonazepam 1 mg three time daily Propranolol 10 mg three times daily Melatonin 10 mg once daily at bedtime | 50-20 mg |
| 16 | 2.75^a^ | "Seen in clinic for dizziness and blurring vision. Denies any chest pain, N/V, fever or diarrhea." – *1 week after XT initiation, on 50-20 mg dose.*  "Referred to clinic for experiencing diarrhea for the past 2 days. Had a large loose bowel movement, one per day. Denies abdominal pain, N/V and appetite is good." – *1 month after XT initiation, 2 weeks after dose increase to 100-20 mg.* | Risperidone 4 mg twice daily Clozapine 100 mg BID (later tapered and discontinued) Bupropion SR 150 mg once daily Buspirone 10 mg three times daily Clonazepam 2 mg three times daily Mirtazapine 45 mg once daily at bedtime Benztropine 0.5 mg twice daily Docusate 100 mg twice daily Glycopyrrolate 2 mg twice daily Ketoconazole shampoo applied once daily Multivitamin once daily Propranolol 20 mg twice daily Vitamin D3 1,000 IU once daily | 100-20 mg |
| 17 | 1 | “Discontinued due to concerns of behavioral disinhibition and increased agitation.” – *2 days after XT initiation on 50-20 mg dose.* Restarted one week later (continued having episodes of agitation the week after discontinuation).   “Patient was invited to treatment team and interview had to be terminated because he began urinating on himself despite having just used the restroom. This was the first time the prescriber had seen him incontinent as they spoke. He continues to wet the bed but doesn't seem concerned and sleeps in a wet bed throughout the night.” - *2 weeks after XT initiation, on 100-20 mg dose.*  “The patient reported having difficulty speaking more clearly because of the XT…ongoing episodes of incontinence and dad continuing to report concerns of appearing overmedicated/oversedated.” – *1 month after XT initiation, on 100-20 mg dose.*  "Unclear if it [XT] made him worse but it certainly did not make him any better." – *Documentation after XT discontinuation.* | Chlorpromazine 200 mg three times daily Invega Sustenna 234 mg IM once every 28 days Depakote DR 750 mg twice daily Clonazepam 2 mg three times daily Diphenhydramine 25 mg twice daily Sodium chloride 1 g four times daily | 100-20 mg |
| 18 | 1.5 | N/A | Aripiprazole 5 mg once daily Clozapine 100 mg once daily at bedtime (tapered within 3 days of initiating XT) Depakote DR 750 mg twice daily Benztropine 1 mg twice daily Diphenhydramine 50 mg once daily at bedtime Calcium carbonate 1,000 mg once daily Docusate 100 mg twice daily Nicotine patch 14 mg applied once daily Propranolol 20 mg three times daily Docusate-senna 2 tab once daily Vitamin D3 2,000 IU once daily | 100-20 mg |
| 19 | 0.5 | "She reported a couple of episodes of vomiting and increased salivation…'I'm spitting and feeling dizzy, why are you putting me on this medication?’" – *1 week after XT initiation, on 100-20 mg dose.*  "There has been significant improvement in hypersalivation and drowsiness and grogginess and she was in a much better mood. She is still fixated on hyperreligious beliefs and has been slightly more hypersexual. Talks about being owner of this facility. Continues to make irrational, grandiose and delusional statements. Continues to refuse routine labs and vital checks. We have not had any success in managing her psychosis thus far." – *1 week after XT discontinuation.* | Haloperidol 10 mg twice daily Haloperidol decanoate 300 mg IM every 28 days Olanzapine 10 mg twice daily Depakote DR 1 g twice daily Clonazepam 0.5 mg twice daily Lorazepam 1 mg twice daily Trazodone 50 mg once daily at bedtime Diphenhydramine 25 mg twice daily Docusate 100 mg twice daily Ferrous sulfate 324 mg twice daily with meals Fish oil 1g twice daily Insulin glargine 20 units daily Insulin lispro sliding scale with meals Metformin 500 mg twice daily Metoprolol XL 25 mg once daily Multivitamin once daily Miralax 17 g once daily Sitagliptin 100 mg once daily Vitamin E 400 IU twice daily | 100-20 mg |
| 20 | 2.25 | "Appears to be tolerating Cobenfy without side effects but it has not been beneficial.” – *2 months after XT initiation, on 125-30 mg dose.* | Invega Sustenna 234 mg IM every 4 weeks Depakote DR 500 mg in the morning, 1,000 mg at bedtime Clonazepam 0.5 mg twice daily Amantadine 100 mg once daily Propranolol 20 mg twice daily Multivitamin once daily Bisacodyl 5 mg once daily Miralax 17 g once daily | 125-30 mg |

Scheduled psychotropic medications are listed at the top, followed by scheduled medications for physical health, followed by as-needed (PRN) medications the patient was charted as receiving during the time they were receiving XT. For medications that indicate they were later tapered/discontinued, the medication order is written as it was prescribed at the time of XT initiation prior to tapering/discontinuing during the patient’s XT treatment course. N/A indicates either adverse effects were not documented, or the notes indicated the patient denied side-effects.
DR: delayed release; ER: extended release; N/V/D: nausea/vomiting/diarrhea; N/V: nausea/vomiting; ODT: orally disintegrating tablet.
^a^Still taking XT at time of chart review.
